# A System of Practical Medicine by American Authors

**Published:** 1885-05

**Authors:** 


**A System of Practical Medicine by American Authors.** Edited by WM. PEPPER, M. D., LL. D., Provost and Professor of Theory and Practice of Medicine and of Clinical Medicine in the University of Pennsylvania. Vol. i. Pathology and General Diseases. Philadelphia: Lea Brothers & Co. 1885.

On the reception of the announcement of this work we predicted, from our knowledge of the editor and the distinguished men associated with him in its production, that it would, upon its completion, be a worthy presentation of the whole field of medicine as it is actually taught and practiced by its best representatives in America. Both editors and publishers are to be congratulated upon the appearance of the first volume. If the succeeding volumes are up to the standard which is reached by the present one, the complete work will be the most valuable and important American medical publication ever issued. As such there is a fitness that it should appear during the centennial year of the existence of the great publishing house, whose imprint it bears. Twenty-one authors contribute to the present volume. The first 227 pages are devoted to general pathology and sanitary science, the remainder of the volume is devoted to the "General Diseases—from special morbid agents operating from without." Dr. Hutchinson, of Philadelphia, writes upon continued fever, typhoid and typhus; Prof. Pepper gives an excellent article upon relapsing fever; Prof. Hyde, of Chicago, contributes articles upon variola varicella and erysipelas, and Prof. White, of Boston, treats of leprosy. Dr. Jacobi's article upon diphtheria, and Dr. I. Lewis Smith's upon scarlet fever, are both able and exhaustive. Prof. Stille's articles upon cholera will be read with interest at the present time. Other subjects treated upon are rubeola and rotheln, malarial fevers, parotitis, yellow fever, plague epidemic, cerebro-spinal meningitis, pertussis, influenza, rabies and hydrophobia, glanders and farcy anthrax, pyæmia and septicæmia, puerperal fever and beriberi. Though all of the articles are not of equal value, all are good. We would most heartily advise all our readers to become subscribers to the work, which we are confident will be one of practical utility.

